# Supplementary material for: Quinagolide Treatment Reduces Invasive and Angiogenic Properties of Endometrial Mesenchymal Stromal Cells
Source: Int J Mol Sci. 2022 Feb 4;23(3):1775. doi: 10.3390/ijms23031775 (PMC8836593; doi:10.3390/ijms23031775)
Supplement: Supplementary file 1 [file ijms-23-01775-s001.zip › ijms-1556619-SI.pdf]

Quinagolide treatment reduces invasive and angiogenic properties of Endometrial Mesenchymal Stromal Cells

Corinne Iampietro[1]*, Alessia Brossa[1]*, Stefano Canosa[2], Stefania Tritta[1], Glenn E Croston[3], Torsten M. Reinheimer[4], Filippo Bonelli[1], Andrea Carosso[2], Gianluca Gennarelli[2], Stefano Cosma[2], Chiara Benedetto[2], Alberto Revelli[2], Benedetta Bussolati[1$]

SUPPLEMENTARY DATA

Supplementary Figure S1

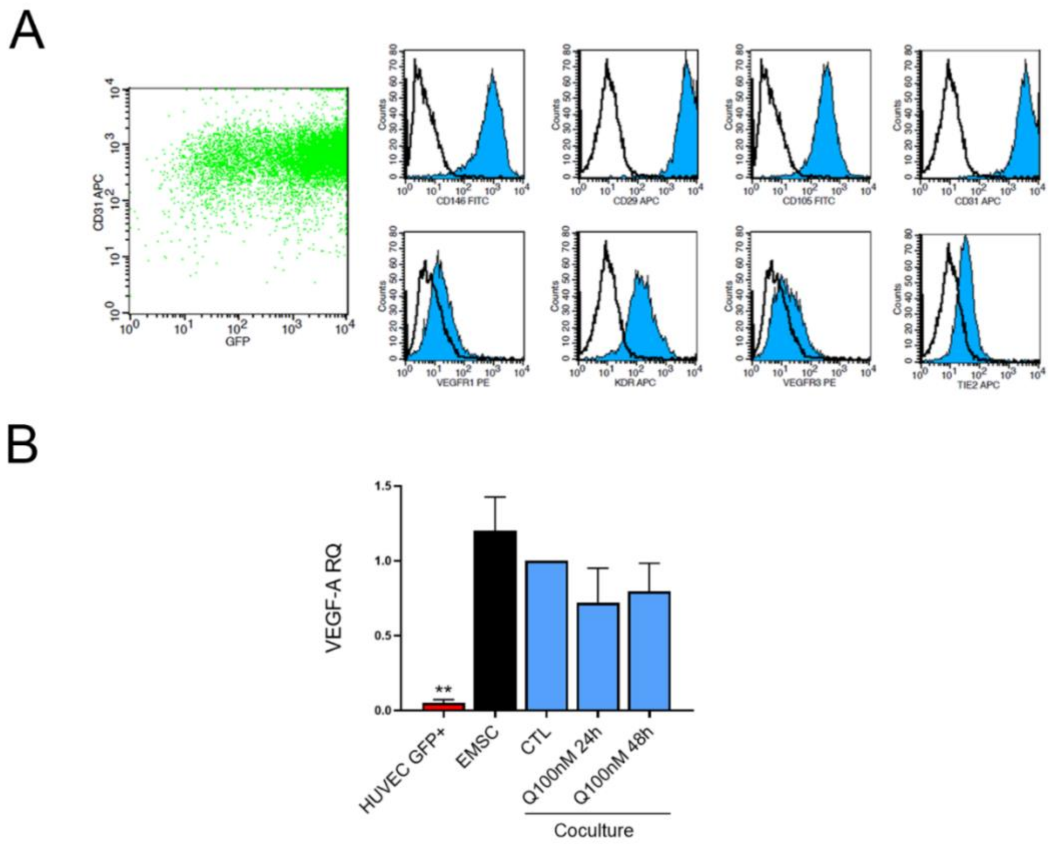

Supplementary Figure S1. Phenotype of GFP^{+} HUVECs and effect of quinagolide on VEGF-A expression by EMSC-HUVEC coculture. A: Representative flow cytometry analysis of HUVEC GFP^{+} cells, showing the expression of GFP and the typical HUVEC markers (CD146, CD29, CD105, CD31, VEGFR1, VEGFR2, VEGFR3 and TIE2). **B:** Real time PCR analysis showing the relative quantification (RQ) of VEGF-A in HUVECs, EMSCs at basal levels and after 48 h co-culture with HUVECs (Coculture, CTL), treated for 24 h or 48 h with 100 nM quinagolide (Q100nM 24h and Q100nM 48h, respectively). No significant effect of quinagolide was observed. Data are expressed as mean ± SD of VEGF-A RQ, normalized to GAPDH and to CTL, of three independent experiments, performed on different EMSC lines. A-Nova was performed: **=p<0.001 respect to CTL.
